# Research and Application of an Air Quality Early Warning System Based on a Modified Least Squares Support Vector Machine and a Cloud Model

**DOI:** 10.3390/ijerph14030249

**Published:** 2017-03-02

**Authors:** Jianzhou Wang, Tong Niu, Rui Wang

**Affiliations:** School of Statistics, Dongbei University of Finance and Economics, Dalian 116025, China; wangjz@dufe.edu.cn (J.W.); wangrui599@tom.com (R.W.)

**Keywords:** air quality, early warning system, forecasting, comprehensive evaluation

## Abstract

The worsening atmospheric pollution increases the necessity of air quality early warning systems (EWSs). Despite the fact that a massive amount of investigation about EWS in theory and practicality has been conducted by numerous researchers, studies concerning the quantification of uncertain information and comprehensive evaluation are still lacking, which impedes further development in the area. In this paper, firstly a comprehensive warning system is proposed, which consists of two vital indispensable modules, namely effective forecasting and scientific evaluation, respectively. For the forecasting module, a novel hybrid model combining the theory of data preprocessing and numerical optimization is first developed to implement effective forecasting for air pollutant concentration. Especially, in order to further enhance the accuracy and robustness of the warning system, interval forecasting is implemented to quantify the uncertainties generated by forecasts, which can provide significant risk signals by using point forecasting for decision-makers. For the evaluation module, a cloud model, based on probability and fuzzy set theory, is developed to perform comprehensive evaluations of air quality, which can realize the transformation between qualitative concept and quantitative data. To verify the effectiveness and efficiency of the warning system, extensive simulations based on air pollutants data from Dalian in China were effectively implemented, which illustrate that the warning system is not only remarkably high-performance, but also widely applicable.

## 1. Introduction

### 1.1. Motivation

With the high-speed growth of the industrial economy in the past decades, atmospheric pollution has been acknowledged as one of the most serious environmental issues, because it not only threatens environmental security, but also induces adverse effects on health [1,2]. Additionally, particulate matter (PM) can also cause many environmental problems such as corrosion, soiling, damage to vegetation and reduced visibility [3]. Accordingly, modeling, forecasting and evaluating air quality play a significant and pivotal part in the early management and warning. However, although they are very vital, relevant studies regarding air quality forecasting and evaluation are still insufficient. High-efficiency forecasting for air quality has the capability to aid the public take effective initiatives to address air pollution, which can reduce the risk of falling ill and enhance living standards. Additionally, scientific evaluation of forecasting results is also an effective means to foresee the diversification of air quality levels. The assessment of air quality is a multiple criteria decision-making process, which can achieve a qualitative evaluation via addressing quantitative information. Effective forecasting and evaluation for air quality can also provide remarkable information to government policymakers to draw up scientific emission policies. Given the aforementioned analysis, a scientific early warning system (EWS) for air quality is urgently needed.

### 1.2. Literature Review

There are a variety of tools that are used to forecast air pollutants concentration, which can be classified into two major models: deterministic models and empirical models [4]. The prevalent deterministic models are chemical transport models (CTMs), which are based on simulating the special mechanisms of atmospheric physics and chemistry. The primary studies on CTMs concentrate on the analysis of pollution sources and the transport of chemical species. Different chemical mechanisms, chemical kinetic expressions, reaction rate coefficients, chemical species and gas phase reactions are usually incorporated into very complex models [5]. The accuracy of CTMs is sensitive to the scale and quality of the emissions data used [6], largely stemming from the incomplete knowledge on the sources, dispersion of PM, transport processes and atmospheric chemicals [4]. Accordingly, compared to empirical models, CTMs is less accurate. Empirical models mainly involve multiple linear regression (MLR), autoregressive integrated moving average model (ARIMA), hidden Markov model and artificial intelligence models, which are generally applied in air pollutant forecasting [5,7,8,9]. However, the most prevalent model for air pollutant forecasting is based on the theory of artificial intelligence, which is efficient and accurate in practical application.

Artificial intelligence models that are exploited to forecast air pollutant concentration mainly include some artificial neural networks and intelligent optimization algorithms, which are very prevalent due to their high-performance learning capacity for nonlinear patterns like air pollution. Song et al. applied a cuckoo search optimization algorithm to optimize the parameters of Weibull, Rayleigh, Lognormal and Gamma distribution functions, which can be conducive to implement interval forecasting further. Besides, the paper applied an adaptive neuro-fuzzy model to perform deterministic forecasting of PM_2.5_ and PM_10_ from three cities in China [9]. Kanchan Prasad et al. developed an adaptive neuro-fuzzy inference system to comprehensively forecast daily air pollution concentrations of five air pollutants, namely SO_2_, NO_2_, CO, O_3_ and PM_10_. In order to reduce the computational cost, a forward selection method was exploited to choose optimal subsets of input dataset [10]. Hybrid artificial intelligence models are more effective and robust than single models. Qin et al. built a hybrid model combining ensemble empirical mode decomposition (EEMD), cuckoo search (CS) and a back-propagation artificial neural network to implement PM forecasting, and the simulation revealed that the hybrid outperformed the benchmark models mentioned in the paper [11]. Niu et al. proposed a novel hybrid decomposition-and-ensemble model based on complementary ensemble empirical mode decomposition (CEEMD), grey wolf optimizer and support vector regression (SVR) to perform PM_2.5_ forecasting, and the empirical study illustrated that the proposed hybrid forecasting model was significantly superior to the benchmark models used in the paper [12]. Zhou et al. presented a general regression neural network (GRNN) model combining EEMD. The function of EEMD is exploited to decompose raw PM_2.5_ data into some intrinsic mode functions (IMFs), and the GRNN is implemented to forecast each IMF. The simulations showed that the developed hybrid EEMD-GRNN model outperformed a single GRNN model without EEMD, MLR model, a principal component regression model, and an ARIMA model [13].

The aforementioned literature on air pollutants forecasting was mainly focused on PM_2.5_ and PM_10_ forecasting, whereas none of them perform comprehensive air pollutants forecasting. In this paper, a comprehensive air pollutants forecasting involving PM_2.5_, PM_10_, O_3_, CO, NO_2_, SO_2_ was carried out. Additionally, most of the aforementioned literature was focused on deterministic forecasting actualized by individual or hybrid models, while few studies implement interval forecasting for air pollutants.

Although the forecasting module has vital significance for EWS, the evaluation system for air quality also plays a remarkable part in air quality EWSs. Scientific evaluation of air pollutants will provide valid information for supervisory departments, and aid them to formulate scientific policies. Many researchers have focused on effective assessment models for air quality. Amit et al. explored a fuzzy—analytical hierarchical process (AHP) model for fuzzy air quality health indexes, which can be used as a signal to reduce health risk [14]. Zhao et al. put forward a fuzzy comprehensive model combined with entropy theory for air quality evaluation, and the model was utilized to address the issue of air quality assessment in Fuxin city [15]. Olvera-García et al. proposed a novel assessment model utilizing fuzzy inference integrated with an analysis hierarchy process, contributing to a new air quality index. Simulation results illustrated that the presented air quality index provided a better evaluation than those in previous studies [16]. The aforementioned evaluation methods have no capability to take the quantification of evaluation factors and the randomness and fuzziness of hierarchy into consideration simultaneously, which makes evaluation results lack relative accuracy. However, a cloud model can achieve the unity between randomness for air quality evaluation and fuzziness for qualitative expression of language.

### 1.3. Aim and Contributions

**Figure 1:**
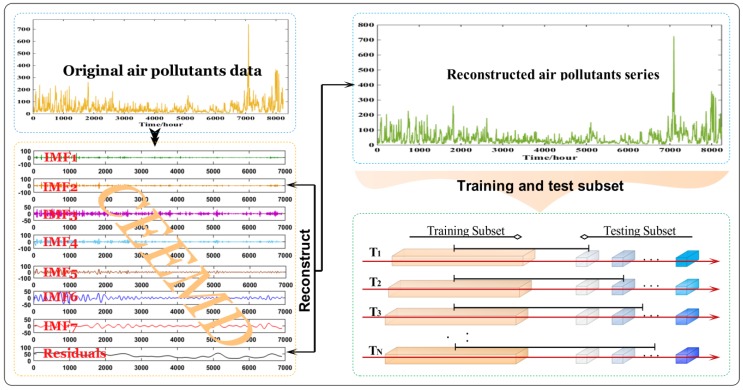
Data preprocessing for EWS.

In the EWS, we designed two novel models to implement point forecasting and interval forecasting for six air pollutants, respectively. For point forecasting, a hybrid model based on the theory of complementary ensemble empirical mode decomposition (CEEMD) and least squares support vector machine (LSSVM) optimized by a modified biogeography-based optimization was successfully proposed, which was designated as CEEMD-BBODE(i.e., a combination of BBO and DE algorithms)-LSSVM. For interval forecasting, a novel interval forecasting model based on the theory of bias and variance estimation and LSSVM regression was developed for interval forecasting, which can overlook the uncertainty of future air pollutant levels and greatly reduce the probability of improper decision-making. Additionally, most papers either involve forecasting or assessment for air quality, whereas studies concerning both forecasting and comprehensive evaluation are very scarce. This paper not only implements air pollutant forecasting but also performs a comprehensive evaluation applying the theory of probability and fuzzy set, forming a novel air quality warning system. The primary step of the proposed EWS can be divided into three steps: firstly, as shown in Figure 1, the original data is decomposed into some intrinsic mode functions (IMFs) by CEEMD, and the first IMF (IMF_1_) that possesses noise feature will be removed. Then, the preprocessed data will be reconstructed into training set and validation set. Secondly, CEEMD-BBODE-LSSVM model and interval forecasting model will be testified by the aforementioned training and validation set. Finally, cloud model will be established on the basis of air quality index and its tiered standards, and then the results of point forecasting for six air pollutants will be regard as an evaluation sample for a cloud model. After 2000 instances of numerical simulation, the final degree of certainty that a sample belongs to certain air quality rating will be determined by averaging the degrees of certainty generated by 2000 simulations. Summarizing, the main contributions of this paper are as follows:

(1) A comprehensive warning system is developed firstly, which consists of a forecasting module and an evaluation module. It is proven as a remarkably effective and high-performance warning system via many numerical implementations;
(2) In the forecasting module, interval forecasting, which has capability to provide more effective and credible information than point forecasting, is implemented effectively;
(3) A modified optimization based on the theory of biogeography is utilized to determine the optimal parameters in LSSVM in order to achieve excellent forecasting performance in the warning system;
(4) A comprehensive evaluation based on probability and fuzzy set is implemented in the EWS, which has enough capability to realize the transformation between qualitative concept and quantitative data.

The remainder of the paper is organized as follows: Section 2 introduces the related methodology utilized in this paper. In Section 3, modeling preparation is reported, and a detail case study that includes point forecasting, interval forecasting and comprehensive evaluation for air quality is effectively implemented. The forecasting effectiveness, implications and future considerations for the EWS are discussed in Section 4. Finally, the conclusions are put forth in the final section.

## 2. Methodology

In this section, the related methodologies of the comprehensive warning system are introduced. Modified optimization based on the theory of biogeography is utilized to optimize the parameters of five distributions for six air pollutants. As for the forecasting module, a hybrid model combining a novel decomposition means, a modified optimization and a classical LSSVM model is developed to implement point and interval forecasting for air pollutants. Additionally, in order to obtain qualitative conclusions about the forecasting results, we apply the evaluation based on the probability and fuzzy set theory to perform an overall assessment of air quality.

### 2.1. Distribution Functions

Statistical distribution functions were utilized to determine the basic characteristics of air pollutant concentration, from which we can penetrate into the uncertainty of air pollutants. Five distribution functions, namely Weibull, Gamma, Lognormal, Log-logistic and Inverse Gaussian were exploited to study the statistical properties of six air pollutants, which are PM_2.5_, PM_10_, O_3_, CO, NO_2_, SO_2_ respectively. The probabilistic distribution functions (PDF) and the cumulative distribution functions (CDF) of the aforementioned distributions are as shown in the Appendix A.

### 2.2. CEEMD

The empirical mode decomposition (EMD) is an adaptive time-frequency data analysis method designed for nonlinear and nonstationary signal analysis [17]. However, the mode mixing problem, a serious deficiency of the EMD, leads to its limitation in practical applications. As a consequence, many modified EMD methods devoted to signal decomposition were developed by researchers [18,19,20,21,22]. The ensemble EMD (EEMD) was developed as a noise-assisted mean, which can thoroughly eliminate the shortcomings of EMD. Time consumption in the process of analyzing large ensemble means and suffering from the residual of the added white noise are remarkable deficiencies in EEMD, even though EEMD has the capability to address the problem of mode mixing effectively. In order to remove these inherent defects of EEMD and improve its calculation efficiency, CEEMD was established by Yeh et al. [23]. As a noise-improved method, the CEEMD not only overcomes the mode mixing problem, but also eliminates the residual added white noise persisting into the IMFs and enhances the calculation efficiency of the EEMD method [24]. In order to eliminate the weaknesses in EMD and EEMD, the CEEMD appends a pair of white Gaussian noises to the original signal, which can make the algorithm save more computing time and lessen the final white noise residue at the same time. The essential steps of CEEMD are as follows:

(1) Given that a single white noise has no enough capability to solve all intermittent signals, we established a positive mixture *f*_1_(*t*) and a negative mixture *f*_2_(*t*) via appending a pair of white noise ($\pm \varepsilon_{n}(t)$) to the original signal:
  (1) $$\{\begin{array}{l} f_{1}(t)=f(t)+\varepsilon_{n}(t)\\ f_{2}(t)=f(t)-\varepsilon_{n}(t) \end{array}$$
(2) Afterward, *k_ij_*^+^ and *k_ij_^−^* are two ensembles of IMFs acquired from decomposing the positive and negative mixtures by the EMD, and *k_ij_*^+^ or *k_ij_^−^* is the *j*th IMF acquired via additive of the *i*th positive noise or negative noise.
(3) Then, the final IMF is computed by:
  (2) $${\mathrm{IMF}}_{j}=\frac{1}{2N}\sum_{i=1}^{N}\left[{k_{ij}}^{+}(t)+{k_{ij}}^{\_}(t)\right]$$
(4) (Accordingly, the original signal *f*(*t*) can be indicated via:
  (3) $$f(t)=\sum_{j=1}^{N}{\mathrm{IMF}}_{j}(t)+r_{n}(t)$$
  where *r_n_*(*t*) is the *n-th* residue (i.e., local trend).

### 2.3. The Modified BBO Algorithm

Biogeography-based optimization (BBO) was originally proposed by Simon [25]. The algorithm stems from a natural process, which can be utilized to address optimization problems in many fields concerning sensor selection [25], power system optimization [26,27], groundwater detection [28] and satellite image classification [29]. The BBO algorithm builds a habitat migration pattern based on probability according to the geographical distribution characteristics of species, in which individuals can probabilistically share information based on a habitat suitability index, and the inferior individuals can be improved by obtaining information from superior individuals. The BBO is an global optimization algorithm that possess powerful exploration capability for the current populations, while its global exploitation capability is poor. On the contrary, differential evolution (DE) possesses commendable exploitation capability, implements effective searches of the decision variable space and can avoid local convergence. To enhance the global exploitation capability of the BBO algorithm, this work proposes a novel modified BBO algorithm in which DE was added to the BBO algorithm when the number of iterations is even, and we designated the modified BBO algorithm as BBODE algorithm, which is essentially a combination of a BBO algorithm and a DE algorithm. The detail pseudo-code of our BBODE algorithm can be seen in Appendix A.

Additionally, there are four migration strategies among single islands in the BBODE algorithm, namely, the cosine model, quadratic model, exponential model, linear model, respectively. The linear model is the most commonly used one in practice. In the algorithm test section we discuss what kind of strategy has the most outstanding performance in the global optimization process. This paper provides four migration strategies in detail, which computational formulas are as shown in Equations (4)–(7) respectively:

Cosine model:

(4) $$\{\begin{array}{l} \lambda_{k}=0.5I(1+cos(\frac{k\pi }{n}))\\ \mu_{k}=0.5E(1-cos(\frac{k\pi }{n})) \end{array}$$

Quadratic model:

(5) $$\{\begin{array}{l} \lambda_{k}=I{\left(\frac{k}{n}-1\right)}^{2}\\ \mu_{k}=E*{\left(\frac{k}{n}\right)}^{2} \end{array}$$

Exponential model:

(6) $$\{\begin{array}{l} \lambda_{k}=I*\exp(-\frac{k}{n})\\ \mu_{k}=E*\exp(\frac{k}{n}-1) \end{array}$$

Linear model:

(7) $$\{\begin{array}{l} \lambda_{k}=I(1-\frac{k}{n})\\ \mu_{k}=E*\frac{k}{n} \end{array}$$

where I denotes maximum possible immigration rate, which will occur when there are no species in the habitat. E represents maximum possible emigration rate, which will happen when the habitat reaches its maximum environment capacity. The terms *λ* and *μ* express the probability of immigration and emigration, respectively. *n* denotes the maximum number of species, and *k* represents the number of species on the *k*th island.

### 2.4. LSSVM

Support vector machine (SVM), a significant branch of machine learning, was proposed by Vapnik [30] on the basis of statistical learning theory, and is an effective means to address pattern recognition and classification missions. The LSSVM based on the structural risk minimization principle is an extension of SVM, which applies the linear least squares criteria to the loss function instead of inequality constraints [31]. In fact, the LSSVM, which spends less computation time than SVM in practice, possesses effective capability in forecasting fields. More details on LSSVM can be found in [32].

It is noteworthy that different types of Mercer kernel function will consequentially generate different LSSVM models. Sigmoid, polynomial and radial basis function (RBF) are frequently-used kernel function for LSSVM model. In [33], the RBF is a prevalent choice for the kernel function on account of the fewer parameters to be set and superior capability in application. Accordingly, this work determined the RBF as the appropriate kernel function:

(8) $$K(x_{i},x_{j})=\exp\left\{-{\left\|x_{j}-x_{i}\right\|}^{2}/2\sigma^{2}\right\}$$

Consequently, in this paper the parameters (i.e., *σ*, *γ*) in the LSSVM model were optimized by our modified BBO algorithm to achieve high-performance forecasting.

### 2.5. Interval Forecasting Based on LSSVM

The LSSVM tool not only implements effective point forecasting, but also performs outstandingly in interval forecasting, which has capability to quantify the uncertainty for point forecasting. In this paper, the LSSVM toolbox in MATLAB provided by De et al. (http://www.esat.kuleuven.be/sista/lssvmlab/) was utilized to carry out interval forecasting for air pollutants. The construction of the forecasting intervals are based on the central limit theorem for linear smoothing combined with bias correction and variance estimation. Details of the code of LSSVM for interval forecasting can be obtained from the aforementioned website, and accordingly here we only a brief description on its steps: Step 1: utilize original data to train the LSSVM model based a RBF basis function. Step 2: calculate the smoother matrix for LSSVM. Step 3: compute the conditional basis and conditional variance. Step 4: set up the significance level. Step 5: obtain forecasting intervals for this fixed significance level. More details about interval forecasting using LSSVM can be found in [34].

### 2.6. Normal Cloud Model Applied for Air Quality Evaluation

**Figure 2:**
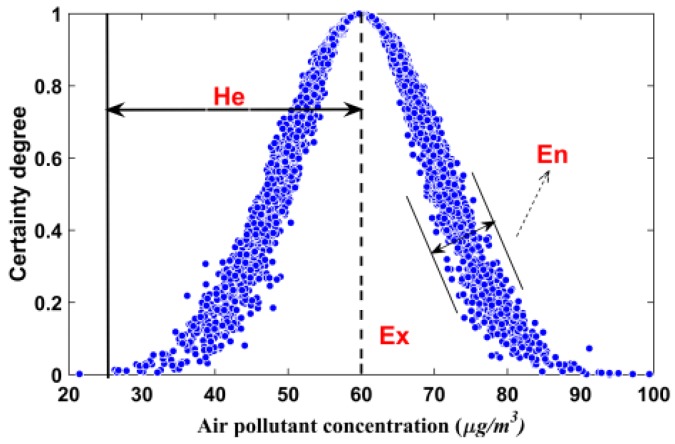
Normal cloud.

A novel hybrid model integrating randomness and fuzziness, namely the cloud model, based on the theory of probability and fuzzy set, presented by Li et al. [35], is an effective cognitive model based on the conversion between qualitative concept and quantitative data, which is applied in many fields. Randomness and fuzziness are generally considered in the evaluation. The cloud model possesses the joint properties of randomness and fuzziness, which are more effective and comprehensive than single randomness or fuzziness model [36]. In Figure 2, the *x*-axis and *y*-axis of normal cloud denote one kind of air pollutant and a certain degree of air quality, respectively. 

**Figure 3:**
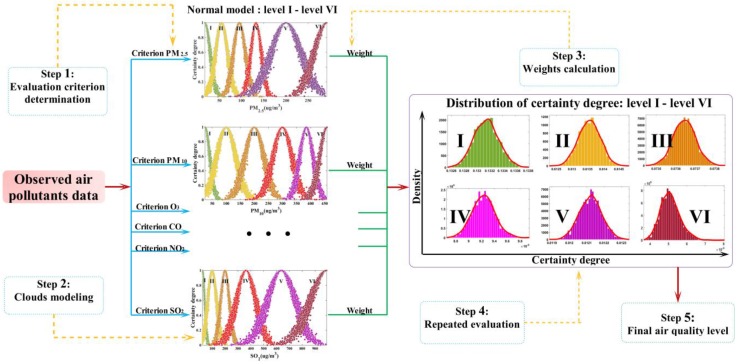
The cloud modeling workflow.

*Ex* denotes the expectation for the quantitative values presenting the level of air quality. *En* indicates the scope of a universe, which can be accepted by the level of air quality. *He* is a measurement for the variation of certainty degree from evaluations. The comprehensive workflow of the cloud model for air quality evaluation is illustrated in Figure 3, and includes five steps.

Determining the air quality criterion (i.e., PM_2.5_, PM_10_, O_3_, CO, NO_2_, SO_2_) is the first step. The second step is to determine the parameters (i.e., *Ex*, *En*, *He*) in the cloud model. The third step is to compute the hybrid entropy, i.e., the analytic hierarchy process (AHP) weights. Transforming the observed data into cloud models repeatedly to achieve the distributions of certainty degrees is the fourth step. The fifth step is to calculate the mean of the certainty degrees and obtain the final air quality level.

The evaluation of air quality is a multi-criteria decision-making process, and the air quality criteria are shown in Table 1. How to properly address steps 2–5 is our primary concern. In this paper, we adopt Equation (9) to compute the cloud model parameters:

(9) $$\{\begin{array}{l} Ex=(B_{max}+B_{min})/2\\ En=(B_{max}-B_{min})/3\\ He=k*En \end{array}$$

where *B_max_* and *B_min_* present the upper bounds and lower bounds of a qualitative concept, which is essentially the grade of an air pollutant criterion. Parameter *k* has the capability to determine the degree of atomization for a normal cloud. Herein, the parameter *k* is supposed as 0.1 to achieve a balance between variation and robustness in the evaluation. It is worthy to note that the *B_max_* of PM_2.5_, PM_10_, O_3_, CO, NO_2_, SO_2_ on the level VI is non-existent. Herein, we utilized a polynomial regression to obtain the pseudo-bounds.

It is significant to emphasize that the half normal cloud model, which is the half of a normal cloud model, was exploited on the highest and lowest level for all criteria, as the certainty degree in this interval is monotonous. As the observed data is beyond the pseudo-bound, the corresponding certainty degree is 1.

The AHP method is widely applied in multi-criteria decision-making processes. Olvera et al. applied the AHP method to estimate the weights (*z_i_*) of PM_2.5_, PM_10_, O_3_, CO, NO_2_, SO_2_ in the evaluation of air quality in Mexico City, which are 0.3, 0.3, 0.233, 0.1, 0.033, 0.033, respectively [17]. However, the AHP method has the inherent deficiency of being sensitive to the potential subjective uncertainty. In order to mitigate the influence of the subjective uncertainty in AHP and regional differences, a hybrid computational method of weights integrating entropy was presented. In the assessment of air quality, the entropy of air pollutant data (*e_t_*) can be computed by Equation (10). Then, the AHP weights based on entropy of *i*th criteria *ω_i_* can be obtained, which is on the basis of normalized entropy (*E_i_*) [37]. Additionally, the *E_i_* and *ω_i_* can be computed by Equations (11) and (12), respectively.

(10) $$e_{t}=-\sum_{t=1}^{T}F_{t}InF_{t}$$

(11) $$E_{i}=\frac{e_{i}}{InT}$$

(12) $$\omega_{i}=\frac{1-E_{i}}{C-\sum_{i=1}^{C}E_{i}}$$

where *F_t_* denotes the frequency of *i*th interval. *e_i_*, namely entropy, represents the uncertainty of observed data for a criterion with *T* intervals. *C* represents the number of criteria.

To balance the latent uncertainty of subjectivity in the AHP method, a novel entropy-AHP method was proposed, which can be calculated via Equation (13). Then, the certainty degree *U* for a level of one criterion can be obtained using Equation (14):

(13) $$W_{i}=\frac{z_{i}\omega_{i}}{\sum_{i}^{C}z_{i}\omega_{i}}$$

(14) $$U=\sum_{i=1}^{C}W_{i}\mu_{i}$$

where *μ_i_* denotes the certainty degree computed by cloud model for each criterion.

## 3. Simulation Modeling and Analysis

In this section, modeling preparations are briefly introduced. A function test is implemented to verify the performance of the BBO and BBODE algorithms. The distribution function parameters for six air pollutants are estimated using BBO and BBODE, respectively. Point and interval forecasting are performed to infer the trends of air pollutants in the future. A comprehensive air quality evaluation is implemented by applying the cloud model.

### 3.1. Modeling Preparations

In this section, the study site, data source and fitness function are briefly described. Six metrics are employed to evaluate the performance of point forecasting and interval forecasting. Finally, a *D-M* test is used to test the forecasting performance.

#### 3.1.1. Study Site and Data Source

In this paper, the Chinese city of Dalian (latitude and longitude 120°58′–123°31′ and 38°43′–40°10′) was selected as the study site for the EWS. It is located in the extreme south of the Liaodong Peninsula. The area of Dalian is 12,573.85 square kilometers. The population of the city is 6.6904 million, and the population density is 464 per square kilometer. In recent years, with the rapid development of the industrial economy of the city, air pollution has been increasingly worsening, which has becomes a growing concern of the public. The deteriorating air quality has increased the incidence of cardiovascular, asthma and lung disease among the public, especially for the elderly and children, which has increased the necessity of an air quality EWS. The existing air quality EWS in the city focuses on monitoring and lacks effective forecasting and comprehensive pollution evaluation, which hinders the development of an effective air quality EWS. Additionally, there is little research on the topic of air quality EWSs in Dalian, and the existing literature puts particular emphasis on cause analysis and air quality indexes, therefore, we chose Dalian as the study site for air quality EWS design.

**Figure 4:**
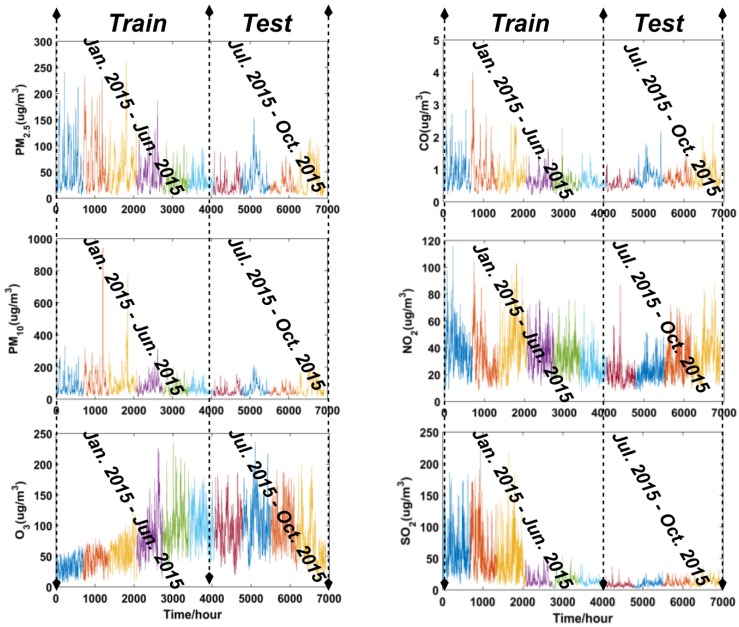
Training and testing subsets for the forecasting model.

The hourly air pollutants data were collected from a website (http://wat.epmap.org/), which is engaged in the collection of environmental data. Data concerning articulate matters (PM_2.5_, PM_10_), ozone (O_3_), carbon monoxide (CO), nitrogen dioxide (NO_2_), sulfur dioxide (SO_2_), as six common air pollutants, were collected from Dalian in the aforementioned website, and were utilized to validate the performance of forecasting models and implement a comprehensive air quality evaluation for the city. Figure 4 shows the study data for the six air pollutants in Dalian, which was divided into a training subset and a testing subset.

#### 3.1.2. The Fitness Function for the CEEMD-BBODE-LSSVM Model

Establishing a proper fitness function is very crucial for the BBODE algorithm, which can build a connection between LSSVM model and the BBODE algorithm and improve the performance of LSSVM via searching for the optimal LSSVM parameters. The fitness function represents the mean of the forecasting error, which is gradually decreasing during the process of searching for the optimal LSSVM parameters until the fitness value satisfies the end condition. In this paper, the fitness function was defined as follows:

(15) $$F=\mathrm{MSE}(\left|y-\hat{y}\right|)$$

where MSE denotes the mean square error between target and forecasting values and *y* and represent the target values and forecasting values, respectively.

#### 3.1.3. The Performance Metric

To determine quantitatively which forecasting model is optimal is our main concern. In this paper, six statistical criteria were utilized to investigate the accuracy and efficiency for point and interval forecasting. Four metrics as shown in Table 2 were used to evaluate the accuracy of point forecasting, which are mean absolute error (MAE), mean absolute percentage error (MAPE), root mean square error (RMSE) and goodness of fit (*R*^2^), respectively. Two criteria were adopted to validate the effectiveness of interval forecasting, which are the coverage probability (CP) and average width (AW), respectively.

CP is a vital metric for interval forecasting, which is evaluated via reckoning the amount of target points within the constructed forecasting intervals. It can verify the effectiveness of interval forecasting with the corresponding significance level (*a*). Theoretically, the forecasting intervals are valid if CP ≥ (1 − *a*)%. If not, the implementation of interval forecasting is invalid. AW provides a measurement of the informativeness for interval forecasting. In theory, the narrow AW can provide greater information value than the wide AW:

(16) $$\begin{array}{l} \mathrm{CP}=\frac{1}{N}\sum_{i=1}^{N}C_{i}\\ i.e.,\;C_{i}=\left\{ \begin{array}{ccc} 1, & if & y_{i}\in [L_{i},U_{i}]\\ 0, & & otherwise \end{array}\right. \end{array}$$

(17) $$\mathrm{AW}=\frac{1}{N}\sum_{i=1}^{N}\left(U_{i}-L_{i}\right)$$

where *L_t_* and *U_t_* represent the lower and upper bounds of the *i*th interval forecasting respectively. *y_i_* denotes target points.

#### 3.1.4. *D-M* Test

The *D-M* test, first proposed by Diebold and Mariano [38], can be utilized to determine whether there is a significant difference among samples. The *D-M* statistic is defined as follows:

(18) $$DM=\frac{\sum_{i=1}^{t}(F({\varepsilon_{t}}^{\left(1\right)})-F({\varepsilon_{t}}^{\left(2\right)}))/t}{\sqrt{S^{2}/t}}S^{2}$$

where *ε_t_*^(1)^ and *ε_t_*^(2)^ denote forecasting errors from two competing models in this paper. Each forecast accuracy is evaluated via an appropriate loss function *F*, and the prevalent loss functions are the square error function and absolute deviation function [39]. *S*^2^ is a variance estimator of $V_{t}=F({\varepsilon_{t}}^{\left(1\right)})-F({\varepsilon_{t}}^{\left(2\right)})$.

The null hypothesis and alternative hypothesis of *D-M* test method are as follows:

(19) $$\begin{array}{l} \mathrm{Null}\;\mathrm{hypothesis},\;H_{0}:E(V_{t})=0\\ \mathrm{Alternative}\;\mathrm{hypothesis},\;H_{1}:E(V_{t})\neq 0 \end{array}$$

In the null hypothesis circumstance, *DM* follows the standard normal distribution *N* (0, 1). The null hypothesis will be rejected if $\left|DM\right|>z_{\alpha /2}$, which means that there is significant difference among samples.

### 3.2. Numerical Analysis of the BBO and BBODE Algorithms

An excellent optimization algorithm should possess the ability of global exploration and local exploitation. To enhance the efficiency of the BBO algorithm, the BBODE algorithm was proposed in this paper. In order to investigate the performance of the BBODE algorithm, the implemented functions tests are described in this section. Six functions as shown in the Appendix A were exploited to validate the capabilities of exploration and exploitation for the BBO and BBODE algorithms. In order to implement an effective and fair comparison between the BBO and BBODE algorithms, each test function was optimized independently 20 times and we initialized random populations in the same way for the different algorithms. The average of the optimal value in each experiment and standard deviation were computed after numerical experiments. All numerical simulations were performed on the platform of MATLAB R2014b for Windows 7 with a 3.30 GHz Intel Core i5, 64 bit CPU and 8 GB RAM. The experimental parameters of BBO and BBODE are shown in Table 3.

The numerical analysis conclusions can be summarized by studying Table 4, which exhibits the results of different test functions with different dimensions, which can sufficiently show that the BBODE algorithm generally has a significant superiority over the BBO algorithm. 

From the detailed information in Table 4, the BBODE algorithm can search for an optimal solution for a sphere function with dimensions of 5 and 10, a Rosenbrock function with dimensions of 2, a Rastrigin function with the dimensions of 2 and 5, a Shaffer function with dimensions of 2, and a Griewank function with dimensions of 2. Considering the elapsed time, BBODE is slightly more time-consuming than BBO. However, considering comprehensively the elapsed time, accuracy and standard deviation, BBODE is still more superior to BBO. Accordingly, the BBODE algorithm was proven to be an efficient and robust optimization algorithm.

**Figure 5:**
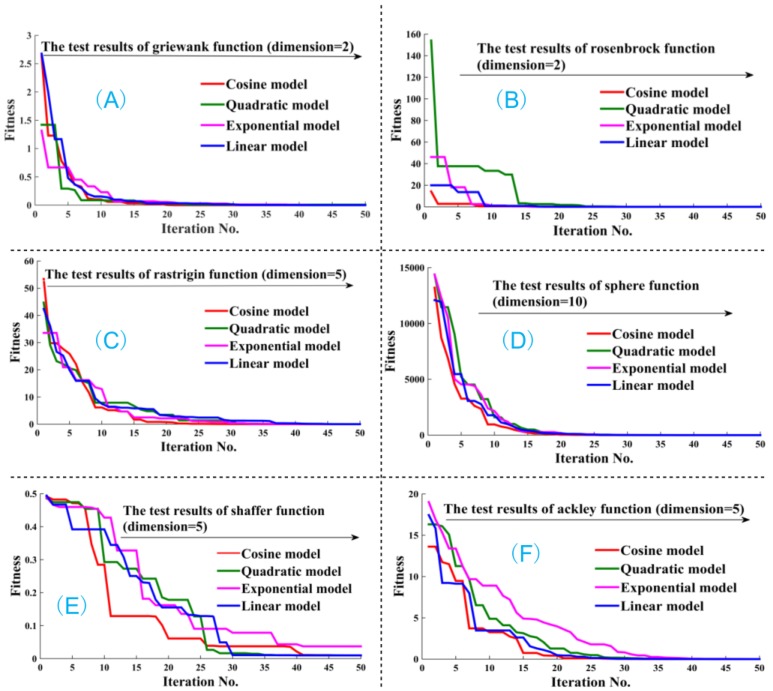
The comparison of convergence speed for four migration strategies in BBODE. In Figure 5, (**A**) four kinds of migration strategies (i.e., cosine model, quadratic model, exponential model, linear model) were tested by griewank function with the dimension 5. From the fitness curve in (**A**), quadratic model has the better convergence speed and accuracy. In (**B**), four kinds of migration strategies were tested by rosenbrock function with the dimension 2. From (**B**), considering convergence speed and accuracy, we can conclude that cosine model has a superior performance. Similarly, in (**C**–**F**), four kinds of migration strategies were tested by rastrigin function with the dimension 5, sphere function with the dimension 10, shaffer function with the dimension 5 and ackley function with the dimension 5, respectively. From (**C**–**F**), compared with quadratic, exponential, linear models, the convergence speed and accuracy of cosine model remarkably illustrate its excellent performance. Summarizing, cosine model is a superior migration strategy in BBODE algorithm.

Additionally, four kinds of migration strategies (i.e., cosine model, quadratic model, exponential model, linear model) in the BBODE algorithm are discussed in this section. Six test functions with different dimensions as shown in Figure 5 are utilized to validate the efficiency of the four strategies. Figure 5 clearly shows that the performance and convergence speed for the four strategies in the migration process, from which it is clearly evident that the cosine model possesses superior performance. Consequently, the cosine model, was adopted as an efficient migration strategy in our BBODE algorithm.

### 3.3. The Distributional Characteristics of the Air Pollutants

Studying the distributional characteristic of air pollutants is an important task, which can reveal the nature and statistical properties of air pollutant data. Six distributions were adopted to perform the analysis of the distribution characteristics of the air pollutants, which are shown in the Appendix A.

The distribution function parameters are commonly estimated by the ways of minimum least square (MLS) and maximum likelihood estimation (MLE). In [9], the experimental results show that artificial intelligent optimization is superior to MLS or MLE in the process of searching for optimal distribution parameters. Accordingly, in this paper, we utilized artificial intelligence optimization to search for the optimal distribution function parameters. 

**Figure 6:**
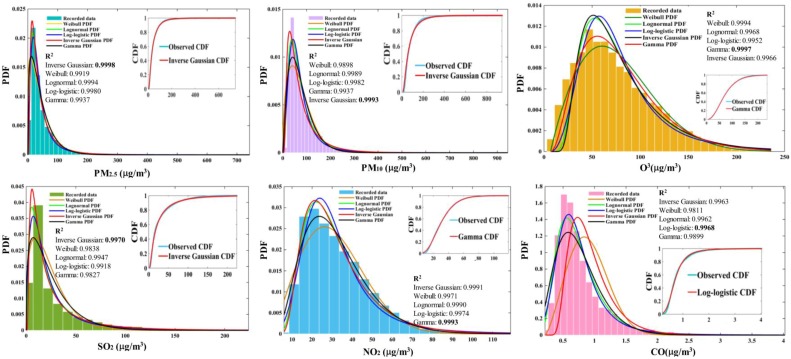
The statistical distribution characteristics of the air pollutant concentrations.

In the function test section, the BBODE algorithm has high performance in the parameter optimization process. Here, the BBODE and BBO algorithms were utilized to search for the optimal distribution function parameters, and we performed a comparison between the performance of the BBODE and BBO algorithms. Table 5 reveals the estimated distribution function parameters obtained for the six air pollutants utilizing the BBODE and BBO algorithms. Goodness of fit (*R*^2^) is adopted to evaluate the fitting performance using different distribution functions and different optimization methods. A larger value indicates better fitting performance. Table 6 presents the *R*^2^ using different artificial intelligent optimization methods, from which can be concluded that the fitting performance using BBODE exceeds the performance of fitting using BBO. Figure 6 shows the combination of frequency histograms and the fitted distributions for six air pollutants. It can be concluded that Inverse Gaussian function performs superior performance in the process of fitting for PM_2.5_, PM_10_, SO_2_ on the reason that the corresponding *R*^2^ is larger than other distributions. The Gamma function is suitable to implement fitting for O_3_ and NO_2_, and Log-logistic distribution is appropriate for fitting the CO data based on the aforementioned reasons.

### 3.4. The Point Forecasting for Air Pollutants

In this section, the proposed hybrid CEEMD-BBODE-LSSVM model was used to implement point forecasting. CEEMD, as a novel decomposition ensemble methodology, was adopted to decompose the original air pollutants data into several IMFs. The parameter setting of CEEMD is as follows: the total number of IMFs and residuals to be decomposed is 8, the standard deviation of added white noise in each ensemble is 0.4, the ensemble number is 200. In actual application, the first IMF will be removed, and the remaining IMFs will be added to construct a new dataset that is used for training and testing the model. The performance of LSSVM is very sensitive to the parameters (i.e., *σ*, *γ*). Therefore, the BBODE algorithm was applied to optimize the parameters in the LSSVM model in order to obtain high-performance forecasting accuracy. The forecasting work was actualized by LSSVM, which is an excellent forecasting tool in many fields. The air pollutants data from Dalian was utilized to test the performance of the proposed hybrid model, which were divided into training subset and testing subset as clearly shown in Figure 4.

**Figure 7:**
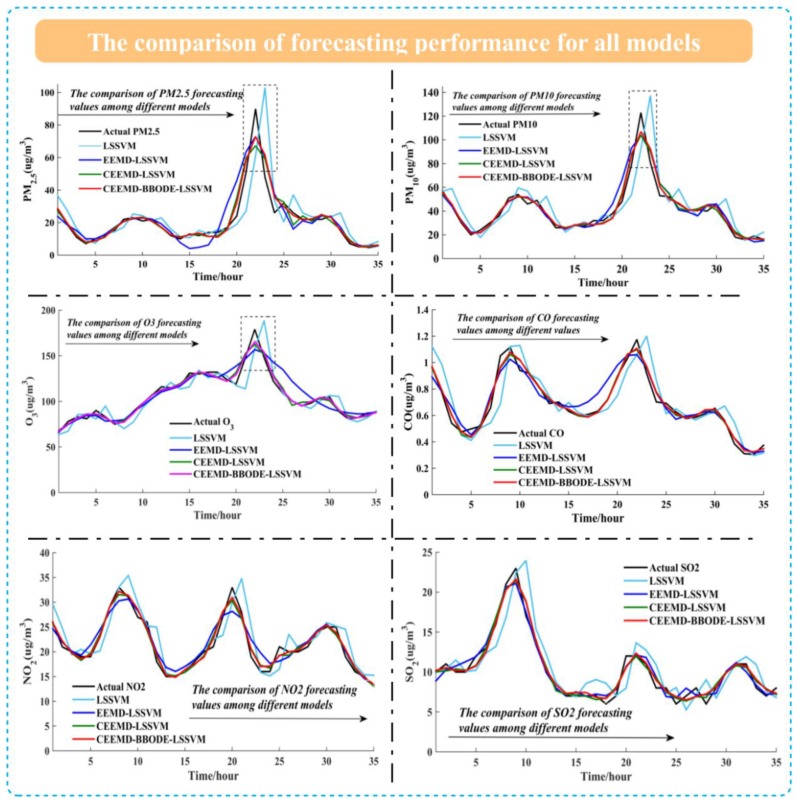
The comparison of forecasting performance for air pollutants in July.

Table 7 and Table 8 report the forecasting performance of all benchmark models for air pollutants from Jul. to Oct. in 2015. Four metrics (i.e., MAE, MAPE, RMSE, *R*^2^) were employed to reveal the forecasting capability for model assessment and comparison. From the forecasting performance of PM_2.5_ in Table 7 and Table 8, the MAE, MAPE, RMSE of LSSVM, EEMD-LSSVM, CEEMD-LSSVM, CEEMD-BBODE-LSSVM are decreasing as a whole, which indicates that CEEMD-BBODE-LSSVM has better performance than the considered benchmark models. The *R*^2^ of LSSVM, EEMD-LSSVM, CEEMD-LSSVM, CEEMD-BBODE-LSSVM for PM_2.5_ forecasting increases progressively, which illustrates that the proposed hybrid model CEEMD-BBODE-LSSVM has superior forecasting capability than the other benchmark models. Similarly, the forecasting performance of CEEMD-BBODE-LSSVM for PM_10_, O_3_, CO, NO_2_, SO_2_ is still superior to that of the other benchmark models. As for decomposition method, compared to models without CEEMD, the models with CEEMD show significant improvements, which illustrates that CEEMD is actually an excellent tool for de-noising. For example, in the forecasting of PM_2.5_, PM_10_, O_3_, CO, NO_2_, SO_2_ in Jul. in Table 7, compared with LSSVM, the MAPE of CEEMD-LSSVM reflects 9.48%, 7.41%, 4.54%, 3.29%, 8.17%, 10.46% improvement, respectively, and the MAPE of CEEMD-LSSVM reflects 2.77%, 1.71%, 1.29%, 0.75%, 1.96%, 2.21% improvement, respectively, compared with EEMD-LSSVM. As for optimization, when making a comparison between CEEMD-LSSVM and CEEMD-BBODE-LSSVM for the six air pollutants in Table 7 and Table 8, CEEMD-BBODE-LSSVM indicates an improvement in forecasting accuracy for CEEMD-LSSVM, which denotes the BBODE algorithm has better performance in the application of searching for optimal solutions for forecasting models. The aforementioned comparative analysis demonstrates that the CEEMD-BBODE-LSSVM model is superior to the benchmark models mentioned in this section. In order to be more clearly illustrate the forecasting performance of all models, we selected the first three days in July to make a visualization, which contain 35 test samples for the air pollutants, respectively. Figure 7 exhibits the comparison of forecasting values based on all models, which shows that the proposed hybrid CEEMD-BBODE-LSSVM model is more accurate and robust. From Figure 7, there is strong correlation between PM_2.5_ and PM_10_ on the reason of the similarity of forecasting results. From the black dotted line in Figure 7, it can be concluded that the CEEMD-BBODE-LSSVM model has outstanding capacity for outlier forecasting. Given the superior performance of the hybrid model in different forecasting environments, we concluded that the hybrid forecasting model has comprehensively wider applicability, effectiveness, compatibility.

### 3.5. The Interval Forecasting for Air Pollutants

The quantification of uncertainty, namely interval forecasting, plays a significant part in air quality EWSs, which can provide more credible and dynamic forecasting results. In this paper, the constructed nonsymmetrical forecasting intervals were generated by LSSVM since the point forecasting has weak capability to address the uncertainties in the forecasting process. Quantitative measures (i.e., AW, CP) are commonly used for evaluating the performance of interval forecasting, which are affected by the different significance level settings.

In theory, the constructed forecasting interval is effective if the condition that the CP is larger or equal to its corresponding confidence level is satisfied. Table 9 reports the numerical results of interval forecasting using the metrics CP and AW quantitatively. 

**Figure 8:**
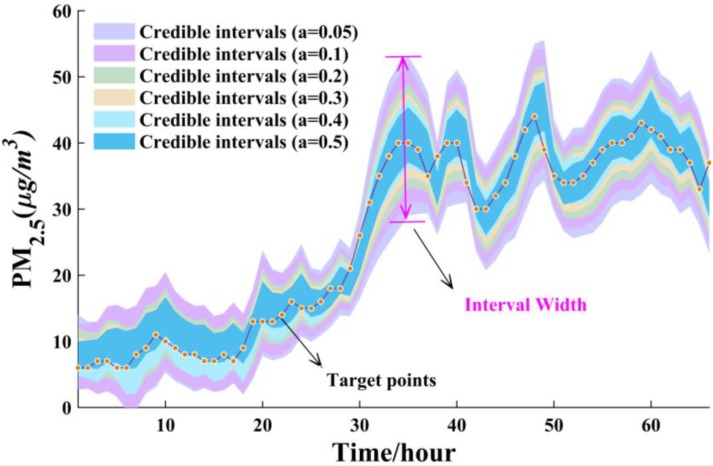
The width of interval forecasting with different significance levels (*a*).

From Table 9, the CP is larger than the corresponding confidence level in most constructed intervals, which remarkably demonstrates that the constructed intervals are valid. It is noteworthy that there is a regular pattern where the interval forecasting width will be smaller when the significance level is increasing gradually, which was displayed schematically in Figure 8 as an illustrative example. The smaller the significance is, the larger the interval forecasting width is. It can observed that the interval forecasting has the best performance when the significance level is 0.05. However, in this situation, it is hard to determine precise values for forecasting when the interval forecasting width is large. The effectiveness of interval forecasting declines when the significance level is increasing. Theoretically, the optimal interval forecasting occurs on actual application and meteorological conditions. For example, the AW can be squeezed if the weather is stable, and AW can be enlarged if the weather is unstable.

**Figure 9:**
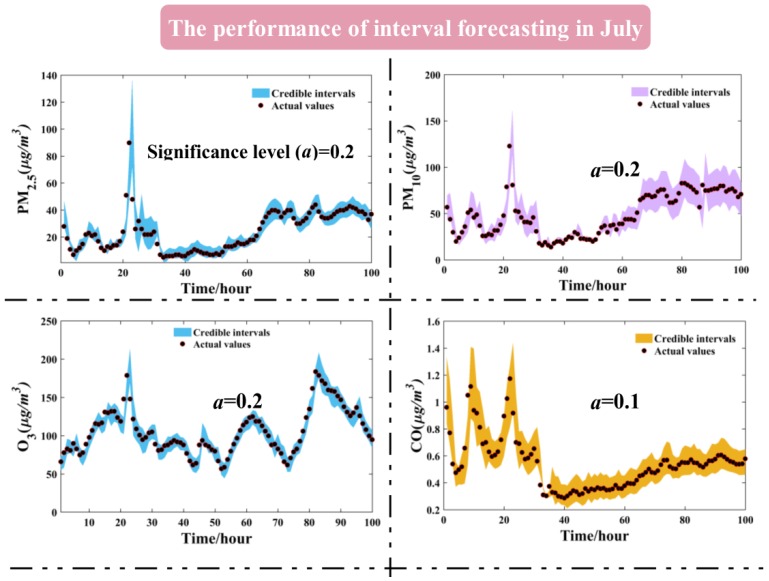

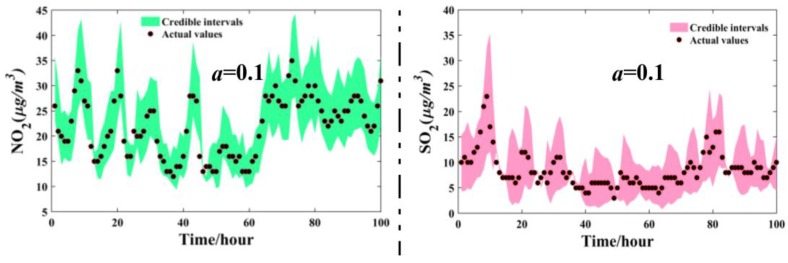
The performance of interval forecasting for all air pollutants in July.

In order to clearly illustrate the interval forecasting results, we adopted the first 100 test samples in July and August to create a visualization, which can be seen in Figure 9. 

In Figure 9, given the informativeness evaluated by CP and correctness assessed by AW in Table 9, we used significance levels of 0.2, 0.2, 0.2, 0.1, 0.1, 0.1 corresponding to PM_2.5_, PM_10_, O_3_, CO, NO_2_, SO_2_ in July to implement interval forecasting, respectively. From Figure 9, it can be observed that most of the actual values are located within the forecasting intervals, which indicates that the efficiency of interval forecasting is theoretically valid. A reference about the hazard using point forecasting will be provided to decision-makers since the uncertainties for forecasting are quantified within the forecasting intervals. Accordingly, the proposed interval forecasting model can provide a tradeoff between effectiveness and informativeness, which is of great importance to formulate scientific policy on early air quality warnings.

### 3.6. Comprehensive Evaluation Implementation

Air quality evaluation is a multiple criteria decision-making process, and the cloud model has outstanding capability to address the fuzziness and randomness in the evaluation process. In this section, a comprehensive evaluation using the cloud model is effectively performed. In the evaluation process, the forecasting values generated by CEEMD-BBODE-LSSVM were regarded as samples to participate in the evaluation, which plays a vital part in EWS.

#### 3.6.1. Evaluation Preparation

Before evaluation, there are some vital sections that need to be prepared, which consist of criteria for air quality, pseudo-boundary for all criteria, parameters in the cloud model, and weights, respectively. The criteria for air quality evaluation are as shown in aforementioned Table 1. The parameters of the cloud model were calculated by Equation (9), and can be seen in Table 10. It is worthy to note that *B_max_* is missing for all level VI criteria, so in this paper we used a polynomial regression to obtain them. The detailed information on the polynomial regression for *B_max_* in level VI for all criteria is shown in Table 11. The weights generated by the hybrid entropy-AHP method for all criteria are reported in Table 12.

#### 3.6.2. Evaluation Implementation

After preparation of the cloud model, a comprehensive assessment was effectively implemented. For the sake of simplicity, we extracted none samples from the testing subset to perform a comprehensive assessment utilizing the cloud model, which is shown in Table 13. 

To enhance the accuracy and robustness, each sample was evaluated over 2000 times, and the mean of the distribution of certainty degree was adopted to determine the final certainty degree. The final air quality levels were attained with the maximum certainty degree, which presents the most possible membership. The final evaluation results for all cases are reported in Table 14. According to aforementioned Table 2, air quality can be classified in six levels: namely excellent, good, light pollution, moderate pollution, heavy pollution, serious pollution. From Table 14, the air quality of A_1_, A_3_, A_7_, A_8_ is at level I. A_2_, A_4_, A_9_ are belong to level II. A_5_ and A_6_ are belong to levels IV and V, respectively. It is worthy to note that the certainty degree 0 in Table 14 indicates that there is no membership at the level.

**Figure 10:**
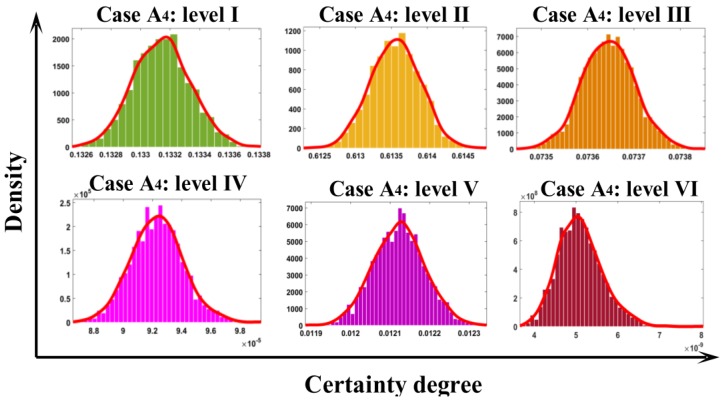
Distributional patterns of certainty degrees in each level of Case A_4_.

In order to illustrate the distribution pattern, we took case A_4_ in Table 13 as an illustrative example. In Figure 10, certainty degrees with different distribution patterns at each level for case A_4_ can be seen. The certainty degree is maximum on the level II for case A_4_, which indicates that case A_4_ belongs to level II. Additionally, when making a comparison among the cases that belong to the same level, more information rather than the simple final level can be provided by the certainty degree. For example, although cases A_2_, A_4_, A_9_ belong to the same level II, their certainty degrees are different. The certainty degree of belonging to level II of cases A_2_, A_4_, A_9_ are 0.5421, 0.6136 and 0.3589, respectively, which allows us to reach the conclusion that case A_4_ is more likely to be level II than cases A_2_, A_9_. The aforementioned discussion revealed that cloud model can not only determine the air quality level, but also further expresses the relative severity of air quality at the same level.

Table 15 shows the *D-M* test results on the basis of MAE loss function, from which a summary can be obtained as follows: in the forecasting of all pollutants, the *D-M* values of LSSVM, EEMD-LSSVM are larger than the upper bound of 1% significance level, which illustrates that CEEMD-BBODE-LSSVM is significantly superior to the LSSVM, EEMD-LSSVM model. Additionally, the *D-M* values for CEEMD-LSSVM are generally larger than the upper bound of 5% significance level, which denotes the proposed CEEMD-BBODE-LSSVM hybrid model has better performance than CEEMD-LSSVM in most cases. Obviously, the proposed hybrid model outperforms other benchmark models generally.

## 4. Discussion

### 4.1. The Forecasting Effectiveness Based on D-M Test

In this paper, a *D-M* test was utilized to distinguish the difference between error series generated by a benchmark forecasting model and a target forecasting model, respectively, which has the capability to verify the point forecasting performance for different forecasting models. 

### 4.2. The Public Health Implications of the EWS

There is few air quality EWS studies in China, which mainly depends on weather research and forecasting models (WRFs). However, WRFs are faced with many challenges in current applications, such as high costs, heavy workload and the difficulty of model debugging in a short time. Additionally, WRFs are usually implemented in the form of grids, and their local forecasting capability is poor. The proposed EWS for Dalian is based on artificial intelligence theory. High precision and scientific evaluation of the EWS in practical application was verified via the aforementioned numerical simulations. The forecasting and evaluation modules in the proposed EWS can be integrated into the existing air monitoring system in Dalian, which will promote the development of an EWS of air quality and provide more warning information for the public. Furthermore, effective warnings about air quality are conducive to lowering the incidence of public health diseases, such as lung, asthma or cardiovascular disease.

### 4.3. Future Considerations for the Air Quality EWS 

In the comparison of EWSs, the factors of effectiveness, efficiency, cost and precision are frequently considered. Although the proposed EWS shows admirable performance in the tasks of forecasting and evaluation, the presented system merely involves empirical models and does not involve the deterministic models mentioned in literature reviews and WRFs. In order to get better performance in the EWS, integration of empirical models, deterministic models and WRFs is necessary in the future, which will combine the respective merits of the three models as much as possible and strengthen the scientific basis of the EWS. Additionally, in order to enhance the practicability of the EWS, it is necessary to establish an information platform on the EWS.

## 5. Conclusions

Establishing a comprehensive air quality warning system plays a particularly crucial role due to the increasing levels of atmospheric pollution. However, how to establish an effective warning system that has best performance is not only a challenging technical assignment, but also a noticeable concern for the public. In this paper, a comprehensive warning system was developed successfully, which consists of effective forecasting and scientific evaluation, respectively. For the forecasting module, a novel hybrid forecasting model, namely CEEMD-BBODE-LSSVM, is proposed for point forecasting. To simplify the complexity of the original data, the series of air pollutants are decomposed into several IMFs using CEEMD, which can be reconstructed by the way of removing high-frequency signals. However, no theory can determine the proper number of IMFs so far, which may be an aspect for future investigations. The BBODE algorithm, as a modified BBO algorithm, is utilized to search for the optimal LSSVM parameters in order to achieve a desirable forecasting performance. The simulation results reveal that the hybrid model is remarkably superior to all benchmark models mentioned on the basis of four metrics (MAE, MAPE, RMSE, *R*^2^). However, point forecasting cannot directly provide the uncertainty information, which means that the decision-maker must bear great risk when using point forecasting. Accordingly, to improve the accuracy and robustness of the forecasting performance, interval forecasting is implemented with the purpose of quantifying the inherent uncertainties, which has the capability to provide malleable information for the future trends of pollutants. Accordingly, it is significant to integrate the point forecasting and interval forecasting, which is essential for optimally regulating air quality. For the evaluation module, air quality is evaluated comprehensively applying a normal cloud model based on entropy–AHP theory, which also plays a vital part in this warning system. Additionally, a multiple dimension cloud model, as an extension of the one dimensional cloud model, is a promising evaluation method, which is a worthy study topic for the future. In this paper, the study of an EWS for air quality is still in a starting phase, which merely involves one-step-ahead forecasting. More exploration on multi-step-ahead forecasting and combination forecasting in theory and practicality should be extensively implemented in the future.

## Figures and Tables

**Table 1:** Quantitative boundaries of air pollution levels of all criteria.

Levels	Air Quality Criteria (µg/m^3^)					
	PM_2.5_	PM_10_	O_3_	CO	NO_2_	SO_2_
I	≤35	≤50	≤10	≤2	≤40	≤50
II	≤75	≤150	≤160	≤4	≤80	≤150
III	≤115	≤250	≤215	≤14	≤180	≤250
IV	≤150	≤350	≤265	≤24	≤280	≤475
V	≤250	≤420	≤800	≤36	≤565	≤800
VI	>250	>420	>800	>36	>565	>800

**Table 2:** Three metric rules for point forecasting.

Metric	Definition	Equation
MAE	Mean absolute error	$\mathrm{MAE}=\frac{1}{n}\sum_{i=1}^{n}\left|y_{i}-y_{i}^{\prime }\right|$
MAPE	Mean absolute percentage error	$\mathrm{MAPE}=\frac{1}{n}\sum_{i=1}^{n}\left|\frac{y_{i}-y_{i}^{\prime }}{y_{i}}\right|\times 100\%$
RMSE	Root mean square error	$\mathrm{RMSE}=\sqrt{\frac{1}{n}\sum_{i=1}^{n}{\left(\frac{y_{i}-y_{i}^{\prime }}{y_{i}}\right)}^{2}}$
*R*^2^	Goodness of fit	$R^{2}=1-\frac{\sum_{i=1}^{n}{\left(y_{i}-y_{i}^{\prime }\right)}^{2}}{\sum_{i=1}^{n}{\left(y_{i}-\bar{y}\right)}^{2}}$

*y_i_* and $y_{i}^{\prime }$ denote the actual values and forecasting values, respectively. represents the average of actual values. The *R*^2^ was also utilized to evaluate the fitness performance in the process of distribution fitting, where *y_i_*, $y_{i}^{\prime }$ and represent the observed cumulative probability, estimated cumulative probability and the average of the observed cumulative probability, respectively.

**Table 3:** The experiment parameters of BBO and BBODE.

Parameter Setting	BBO	BBODE
Maximum iteration	5000	5000
Population size	50	50
The number of elite kept	3	3
Maximum emigration rate	1	1
Minimum emigration rate	0	0
Maximum immigration rate	1	1
Minimum immigration rate	0	0
Mutation probability	0.05	0.4
Difference operator	-	0.6

**Table 4:** Test results of BBO and BBODE.

Test Function	Dimension	Algorithm	Optimal/Worse Solution	Mean/Std.	Elapsed Time (s)
Sphere	5	BBO	3.83 × 10^−3^/1.87 × 10^−2^	1.21 × 10^−2^/6.09 × 10^−3^	24.5293
		BBODE	0/0	0/0	25.1026
	10	BBO	1.05 × 10^−2^/3.42 × 10^−1^	8.06 × 10^−2^/3.14 × 10^−2^	27.1782
		BBODE	0/0	0/0	28.0055
Rosenbrock	2	BBO	1.05 × 10^−2^/6.19 × 10^−1^	2.65 × 10^−1^/2.48 × 10^−1^	21.5151
		BBODE	0/0	0/0	38.8187
Rastrigin	2	BBO	1.56 × 10^−4^/3.71 × 10^−3^	1.70 × 10^−3^/1.46 × 10−3	22.1951
		BBODE	0/0	0/0	23.0743
	5	BBO	3.97 × 10^−3^/2.05 × 10^−2^	1.15 × 10^−2^/6.43 × 10^−3^	24.4002
		BBODE	0/0	0/0	24.1739
Shaffer	2	BBO	9.72 × 10^−3^/3.33 × 10^−2^	1.45 × 10^−2^/1.06 × 10^−2^	22.4175
		BBODE	0/0	0/0	23.3923
	5	BBO	9.72 × 10^−3^/7.82 × 10^−2^	3.99 × 10^−2^/2.45 × 10^−2^	24.3080
		BBODE	9.72 × 10−3/9.72 × 10^−3^	9.70 × 10^−3^/9.23 × 10^−11^	29.3161
Griewank	2	BBO	3.60 × 10^−3^/6.80 × 10^−2^	2.06 × 10^−2^/2.67 × 10^−2^	22.2211
		BBODE	0/7.40 × 10^−3^	3.00 × 10^−3^/4.05 ×10^−3^	22.4311
Ackley	2	BBO	2.61 × 10^−2^/8.12 × 10^−2^	5.24 × 10^−2^/2.57 × 10^−2^	22.4061
		BBODE	8.88 × 10^−16^/8.88 × 10^−16^	0/0	22.9809
	5	BBO	2.78 × 10^−2^/2.90 × 10^−1^	1.20 × 10^−1^/1.07 × 10^−1^	24.3998
		BBODE	8.88 × 10^−16^/8.88 × 10^−16^	0/0	25.2199

**Table 5:** Parameters of the different distributions based on the different optimized algorithm. In Table 5, *a* and *b* represent scale and shape parameters of distribution functions, respectively.

Indexes	Optimized Algorithm	Parameters									
		Weibull		Gamma		Lognormal		Log-Logistic		Inverse Gaussian	
		*a*	*b*	*a*	*b*	*a*	*b*	*a*	*b*	*a*	*b*
PM_2.5_	BBO	45.6353	1.1494	1.1219	39.3960	0.9590	3.3423	1.9971	31.9631	46.2247	47.6059
	BBODE	45.7883	1.1754	1.3769	31.5937	0.8675	3.4642	1.9865	32.0344	46.1661	47.6248
PM_10_	BBO	82.0580	1.3799	4.5728	15.1932	0.7351	3.9522	2.6477	62.4040	77.6225	137.8372
	BBODE	82.1414	1.5152	2.2187	33.7894	0.6939	4.1205	2.4676	61.5932	78.0797	137.2609
O_3_	BBO	81.9614	1.8979	3.7011	19.7425	0.6156	4.0754	3.0971	65.1357	76.2749	199.8628
	BBODE	82.0189	1.8920	3.0735	24.0945	0.5648	4.1715	3.0136	64.9673	75.8027	213.8432
SO_2_	BBO	27.1355	1.1097	0.9696	28.5482	2.8314	1.0833	1.6724	17.3678	27.1713	19.4500
	BBODE	26.4620	0.9312	0.9194	29.4822	2.8378	1.0554	1.6357	17.1035	29.3444	18.3036
NO_2_	BBO	35.1287	2.1449	3.8436	8.2904	0.5596	3.2623	3.2489	28.1741	32.5209	112.2516
	BBODE	35.2476	2.1360	3.8948	8.1566	0.5075	3.3501	3.3673	28.5397	32.3743	114.6579
CO	BBO	0.8547	2.2424	1.1233	0.7854	0.4562	−0.3797	3.8337	0.7208	1.0952	1.0957
	BBODE	0.8229	2.3983	4.4389	0.1689	0.4558	−0.3789	3.7101	0.6832	0.7589	3.3818

**Table 6:** *R*^2^ of different distribution using different optimized algorithm. The data in bold denotes that it is largest in each line of Table 6, which represents the optimal *R*^2^ of distribution fitting.

Indexes	Optimized Algorithm	Evaluation Criteria (*R*^2^)				
		Weibull	Gamma	Lognormal	Log-Logistic	Inverse Gaussian
PM_2.5_	BBO	0.9918	0.9904	0.9919	0.9980	0.9998
	BBODE	0.9919	0.9937	0.9994	0.9980	**0.9998**
PM_10_	BBO	0.9879	0.9634	0.9760	0.9970	0.9991
	BBODE	0.9898	0.9937	0.9989	0.9982	**0.9993**
O_3_	BBO	0.9984	0.9979	0.9870	0.9950	0.9963
	BBODE	**0.9994**	0.9997	0.9968	0.9952	0.9966
SO_2_	BBO	0.9747	0.9820	0.9944	0.9916	0.9942
	BBODE	0.9838	0.9827	0.9947	0.9918	**0.9970**
NO_2_	BBO	0.9971	0.9990	0.9901	0.9970	0.9991
	BBODE	0.9971	**0.9993**	0.9990	0.9974	0.9991
CO	BBO	0.9774	0.8257	0.9962	0.9894	0.8309
	BBODE	0.9811	0.9899	0.9962	**0.9968**	0.9963

**Table 7:** Performance evaluations of all forecasting models for air pollutants in July and August.

**Jul.**	**LSSVM**				**EEMD-LSSVM**				**CEEMD-LSSVM**				**CEEMD-BBODE-LSSVM**			
	**MAE** **(µg/m^3^)**	**MAPE** **(%)**	**RMSE** **(µg/m^3^)**	***R*^2^**	**MAE** **(µg/m^3^)**	**MAPE** **(%)**	**RMSE** **(µg/m^3^)**	***R*^2^**	**MAE** **(µg/m^3^)**	**MAPE** **(%)**	**RMSE** **(µg/m^3^)**	***R*^2^**	**MAE** **(µg/m^3^)**	**MAPE** **(%)**	**RMSE** **(µg/m^3^)**	***R*^2^**
PM_2.5_	2.7493	13.72	4.6403	0.9190	1.5257	7.01	2.8392	0.9697	0.9223	4.24	1.7455	0.9885	0.8377	3.86	1.5264	0.9912
PM_10_	4.7844	10.87	7.6946	0.9228	2.3329	5.17	3.7108	0.9821	1.5476	3.46	2.5508	0.9915	1.5004	3.34	2.4581	0.9921
O_3_	5.7668	6.81	7.9288	0.9451	3.0425	3.56	4.4377	0.9828	1.9619	2.27	2.7241	0.9935	1.7602	2.04	2.4161	0.9949
CO	0.0282	4.93	0.0461	0.9021	0.0137	2.39	0.0225	0.9766	0.0094	1.64	0.0153	0.9892	0.0093	1.64	0.0150	0.9896
NO_2_	2.4432	12.82	3.6164	0.8298	1.2705	6.61	1.8767	0.9542	0.8877	4.65	1.3733	0.9755	0.8138	4.29	1.2850	0.9785
SO_2_	1.3173	17.17	1.9538	0.7346	0.6607	8.92	0.9071	0.9428	0.5091	6.71	0.7596	0.9599	0.4762	6.42	0.7222	0.9637
**Aug.**	**LSSVM**				**EEMD-LSSVM**				**CEEMD-LSSVM**				**CEEMD-BBODE-LSSVM**			
	**MAE** **(µg/m^3^)**	**MAPE** **(%)**	**RMSE** **(µg/m^3^)**	***R*^2^**	**MAE** **(µg/m^3^)**	**MAPE** **(%)**	**RMSE** **(µg/m^3^)**	***R*^2^**	**MAE** **(µg/m^3^)**	**MAPE** **(%)**	**RMSE** **(µg/m^3^)**	***R*^2^**	**MAE** **(µg/m^3^)**	**MAPE** **(%)**	**RMSE** **(µg/m^3^)**	***R*^2^**
PM_2.5_	2.8102	10.12	4.2173	0.9718	1.3468	4.72	2.0854	0.9931	0.9601	3.35	1.4169	0.9968	0.8584	3.00	1.2814	0.9974
PM_10_	4.6826	8.27	7.9020	0.9517	4.6866	8.32	7.8981	0.9518	1.4682	2.55	2.4766	0.9953	1.4201	2.47	2.4687	0.9953
O_3_	6.8965	7.19	9.2676	0.9454	4.3948	4.66	5.7059	0.9793	2.1932	2.30	2.9572	0.9944	1.9939	2.05	2.6971	0.9954
CO	0.0382	4.83	0.0628	0.9453	0.0196	2.52	0.0350	0.9830	0.0126	1.65	0.0196	0.9946	0.0123	1.63	0.0192	0.9949
NO_2_	2.6935	12.67	3.8906	0.7507	1.7882	8.44	2.5133	0.8960	1.1073	5.11	1.5708	0.9594	0.9906	4.62	1.4239	0.9666
SO_2_	1.4433	16.43	2.0774	0.7876	0.7174	8.49	0.9874	0.9520	0.5274	6.17	0.7691	0.9709	0.5124	6.05	0.7568	0.9718

**Table 8:** Performance evaluations of all forecasting models for air pollutants in September and October.

**Sept.**	**LSSVM**				**EEMD-LSSVM**				**CEEMD-LSSVM**				**CEEMD-BBODE-LSSVM**			
	**MAE** **(µg/m^3^)**	**MAPE** **(%)**	**RMSE** **(µg/m^3^)**	***R*^2^**	**MAE** **(µg/m^3^)**	**MAPE** **(%)**	**RMSE** **(µg/m^3^)**	***R*^2^**	**MAE** **(µg/m^3^)**	**MAPE** **(%)**	**RMSE** **(µg/m^3^)**	***R*^2^**	**MAE** **(µg/m^3^)**	**MAPE** **(%)**	**RMSE** **(µg/m^3^)**	***R*^2^**
PM_2.5_	1.8597	9.26	2.6744	0.9632	0.8473	3.91	1.2725	0.9917	0.5845	2.73	0.8504	0.9963	0.5329	2.55	0.7836	0.9968
PM_10_	3.1881	7.25	4.6004	0.9544	1.4800	3.29	2.0462	0.9910	0.9906	2.15	1.4081	0.9957	0.9464	2.10	1.3310	0.9962
O_3_	6.1037	7.12	8.4823	0.9444	3.8598	4.52	5.5017	0.9766	1.9423	2.30	2.6734	0.9945	1.7936	2.10	2.4833	0.9952
CO	0.0343	4.80	0.0501	0.9231	0.0337	4.70	0.0501	0.9229	0.0108	1.51	0.0159	0.9922	0.0106	1.49	0.0155	0.9926
NO_2_	3.3152	11.05	4.7473	0.8577	2.5733	9.40	3.6797	0.9145	1.3327	4.44	1.9169	0.9768	1.1959	3.99	1.7156	0.9814
SO_2_	1.5666	14.18	2.1999		0.6901	6.46	0.9278	0.9611	0.5627	5.08	0.8056	0.9707	0.5429	4.87	0.7764	0.9728
**Oct.**	**LSSVM**				**EEMD-LSSVM**				**CEEMD-LSSVM**				**CEEMD-BBODE-LSSVM**			
	**MAE** **(µg/m^3^)**	**MAPE** **(%)**	**RMSE** **(µg/m^3^)**	***R*^2^**	**MAE** **(µg/m^3^)**	**MAPE** **(%)**	**RMSE** **(µg/m^3^)**	***R*^2^**	**MAE** **(µg/m^3^)**	**MAPE** **(%)**	**RMSE** **(µg/m^3^)**	***R*^2^**	**MAE** **(µg/m^3^)**	**MAPE** **(%)**	**RMSE** **(µg/m^3^)**	***R*^2^**
PM_2.5_	3.1429	14.75	5.2280	0.9632	2.0940	7.18	3.9670	0.9788	1.0080	4.02	1.8061	0.9956	0.9656	3.87	1.6485	0.9963
PM_10_	5.5187	9.84	8.9370	0.9596	3.3163	5.39	5.7764	0.9831	1.7639	2.98	2.9540	0.9956	1.7107	2.64	2.8270	0.9960
O_3_	5.2873	8.89	7.5633	0.9622	3.1799	5.05	4.6041	0.9860	1.7490	2.99	2.5731	0.9956	1.5749	2.70	2.3123	0.9965
CO	0.0491	6.26	0.0883	0.9185	0.0475	6.09	0.0846	0.9251	0.0173	2.22	0.0329	0.9887	0.0171	2.10	0.0318	0.9895
NO_2_	3.2192	10.74	4.6240	0.9025	2.4256	8.04	3.7272	0.9366	1.2379	4.13	1.8160	0.9850	1.1440	3.81	1.6778	0.9872
SO_2_	1.5912	14.01	2.2161	0.8578	0.7122	6.40	0.9919	0.9715	0.5605	5.01	0.8241	0.9803	0.5541	4.90	0.7995	0.9815

**Table 9:** The evaluation results of interval forecasting using CP and AW.

Indexes		PM_2.5_		PM_10_		O_3_		CO		NO_2_		SO_2_	
	*a*	CP	AW	CP	AW	CP	AW	CP	AW	CP	AW	CP	AW
**Jul.**	0.1	94.26%	13.6618	90.48%	31.0273	93.12%	26.7807	98.60%	0.2079	92.72%	9.9325	90.20%	8.4259
	0.2	90.62%	10.4174	89.92%	24.4988	89.22%	20.7596	97.06%	0.1606	82.21%	7.4009	88.80%	6.6628
	0.3	84.45%	8.3868	86.30%	22.4904	81.79%	16.7552	94.68%	0.1292	76.19%	6.2515	84.03%	5.1160
	0.4	76.47%	6.9376	78.01%	15.5500	74.37%	13.7277	90.03%	0.0911	71.43%	4.9870	79.61%	4.2380
**Aug.**	0.1	94.04%	16.1549	96.78%	37.3582	91.27%	28.6477	98.34%	0.2980	91.83%	10.6406	91.27%	9.8413
	0.2	89.06%	10.4853	95.24%	29.4140	84.90%	22.1802	96.81%	0.2318	83.52%	8.2517	89.61%	7.6514
	0.3	84.49%	10.1968	91.60%	23.3305	76.45%	17.9500	94.74%	0.1849	78.39%	6.9446	86.70%	6.1226
	0.4	80.03%	8.1918	88.39%	19.2456	68.42%	14.6038	91.74%	0.1390	73.14%	5.0025	82.57%	4.7893
**Sept.**	0.1	97.36%	10.8554	89.52%	27.8707	91.79%	27.5385	99.27%	0.2646	92.82%	13.6887	90.03%	10.6070
	0.2	95.60%	8.8471	88.08%	22.5034	86.22%	21.4810	98.24%	0.2052	81.97%	10.5548	88.94%	8.0384
	0.3	91.94%	7.0965	87.50%	18.5714	80.21%	17.4530	94.13%	0.1627	79.62%	8.6191	86.07%	6.5208
	0.4	88.21%	6.1263	86.13%	14.7516	72.73%	14.1431	90.38%	0.1328	76.93%	5.9972	83.16%	3.9879
**Oct.**	0.1	94.15%	17.7601	92.72%	39.6436	92.89%	23.2962	96.03%	0.3322	93.57%	14.7220	94.39%	11.6736
	0.2	90.97%	13.1872	89.64%	31.1285	88.74%	18.0237	92.89%	0.2587	83.88%	11.4790	92.75%	9.2227
	0.3	87.82%	10.9541	83.38%	24.9457	79.34%	14.6879	90.83%	0.2106	81.53%	9.2536	90.01%	7.2835
	0.4	84.43%	7.4732	80.13%	20.6901	72.09%	11.9308	88.86%	0.1884	78.64%	7.0376	87.49%	5.0129

**Table 10:** The parameters of the cloud model for all criteria.

**Levels**	**PM_2.5_**			**PM_10_**			**O_3_**		
	***Ex***	***En***	***He***	***Ex***	***En***	***He***	***Ex***	***En***	***He***
I	17.5	11.67	1.17	25	16.67	1.67	5	3.33	0.33
II	55	13.33	1.33	100	33.33	3.33	85	50	5
III	95	13.33	1.33	200	33.33	3.33	187.5	18.33	1.83
IV	132.5	11.67	1.17	300	33.33	3.33	240	16.67	1.67
V	200	33.33	3.33	385	23.33	2.33	532.5	178.33	17.83
VI	291.99	28.00	2.80	457.95	25.30	2.53	988.8	125.86	12.59
**Levels**	**CO**			**NO_2_**			**SO_2_**		
	***Ex***	***En***	***He***	***Ex***	***En***	***He***	***Ex***	***En***	***He***
I	1	0.67	0.07	20	13.33	1.33	25	16.67	1.67
II	3	0.67	0.07	60	13.33	1.33	100	33.33	3.33
III	9	3.33	0.33	130	33.33	3.33	200	33.33	3.33
IV	19	3.33	0.33	230	33.33	3.33	362.5	75	7.5
V	30	4	0.4	422.5	95	9.5	637.5	108.33	10.83
VI	44.21	5.47	0.55	707	94.67	9.47	989.97	126.65	12.67

**Table 11:** Polynomial regression for *B_max_* of all criteria with level VI.

Indices	Polynomial Regression	*B_max_* of Level VI
PM_2.5_	*f*(*x*) = 8.21*x*^2^ + 1.21*x* + 31	333.99
PM_10_	*f*(*x*) = −4.29*x*^2^ + 119.7*x* − 68	495.91
O_3_	*f*(*x*) = 54.64*x*^2^ − 159.4*x* + 167	1177.6
CO	*f*(*x*) = 1.43*x*^2^ + 0.23*x* − 0.4	52.42
NO_2_	*f*(*x*) = 35*x*^2^ − 85*x* + 99	849
SO_2_	*f*(*x*) = 41.07*x*^2^ − 63.93*x* + 85	1179.94

**Table 12:** AHP-entropy weights for all criteria.

Criteria	AHP Weight *z*	Entropy	Entropy Weight *ω*	Entropy-AHP Weight *W*
PM_2.5_	0.3	4.6692	0.2348	0.4292
PM_10_	0.3	5.0828	0.1917	0.3505
O_3_	0.233	5.1281	0.0621	0.0881
CO	0.1	6.9810	0.0721	0.0439
NO_2_	0.033	4.0407	0.1730	0.0348
SO_2_	0.033	4.1733	0.2662	0.0535

**Table 13:** The forecasting samples from test subset used for evaluation.

Date	PM_2.5_ (µg/m^3^)	PM_10_ (µg/m^3^)	O_3_ (µg/m^3^)	CO (µg/m^3^)	NO_2_ (µg/m^3^)	SO_2_ (µg/m^3^)	Cases
1 July 2015 1:00	28.7706	55.7602	67.3450	0.9697	25.8604	10.2004	A_1_
1 July 2015 23:00	72.9066	107.0337	165.4987	1.1086	20.4798	11.1226	A_2_
2 July 2015 9:00	8.4205	20.8968	82.3072	0.4267	20.5176	10.7273	A_3_
2 August 2015 17:00	47.5483	69.4576	175.0633	0.7634	22.8088	6.7971	A_4_
14 August 2015 20:00	127.6426	178.7458	217.2556	1.1993	25.2613	13.9264	A_5_
15 August 2015 0:00	154.4614	211.3916	228.8058	1.3244	17.0485	16.1272	A_6_
1 September 2015 1:00	17.9037	34.8418	78.1009	0.6857	24.9247	7.6599	A_7_
1 Octorber 2015 8:00	20.5887	37.0588	95.6089	1.0228	21.8282	4.3759	A_8_
5 Octorber 2015 13:00	75.7741	135.5992	157.9036	1.1049	25.8181	21.8493	A_9_

**Table 14:** The final results of evaluation for air quality.

Cases	Final Certainty Degree						Final Air Quality Level
	I	II	III	IV	V	VI	
A_1_	0.4599	0.2930	0.0030	0.0000	0.0033	0.0000	**I**
A_2_	0.1316	0.5421	0.1623	0.0000	0.0115	0.0000	**II**
A_3_	0.9119	0.1142	0.0018	0.0000	0.0040	0.0000	**I**
A_4_	0.1331	0.6136	0.0736	0.0001	0.0121	0.0000	**II**
A_5_	0.1276	0.0308	0.3346	0.4278	0.0608	0.0000	**IV**
A_6_	0.1272	0.0085	0.1554	0.1877	0.3408	0.0000	**V**
A_7_	0.8518	0.1532	0.0025	0.0000	0.0038	0.0000	**I**
A_8_	0.8138	0.1652	0.0029	0.0000	0.0048	0.0000	**I**
A_9_	0.1284	0.3589	0.2323	0.0000	0.0108	0.0000	**II**

**Table 15:** The results of *D-M* test.

***D-M*** **Test**		**Jul.**					
**Benchmark Model**	**Target Model**	**PM_2.5_**	**PM_10_**	**O_3_**	**CO**	**NO_2_**	**SO_2_**
LSSVM	CEEMD-BBODE-LSSVM	4.28249 *	6.99631 *	11.55773 *	6.69119 *	8.35994 *	7.56095 *
EEMD-LSSVM	CEEMD-BBODE-LSSVM	5.43788 *	6.39938 *	7.41868 *	4.92945 *	8.24263 *	5.24603 *
CEEMD-LSSVM	CEEMD-BBODE-LSSVM	2.00715 **	1.81403 ***	5.80117 *	1.65674 ***	2.72935 *	2.51401 **
***D-M*** **Test**		**Aug.**					
**Benchmark Model**	**Target Model**	**PM_2.5_**	**PM_10_**	**O_3_**	**CO**	**NO_2_**	**SO_2_**
LSSVM	CEEMD-BBODE-LSSVM	8.35825 *	5.99979 *	12.10402 *	7.63585 *	8.51850 *	8.87180 *
EEMD-LSSVM	CEEMD-BBODE-LSSVM	6.60765 *	5.96828 *	14.35558 *	4.55709 *	9.98957 *	6.79926 *
CEEMD-LSSVM	CEEMD-BBODE-LSSVM	5.06978 *	0.13336	4.62010 *	1.77389 ***	5.05217 *	0.77962
***D-M*** **Test**		**Sep.**					
**Benchmark Model**	**Target Model**	**PM_2.5_**	**PM_10_**	**O_3_**	**CO**	**NO_2_**	**SO_2_**
LSSVM	CEEMD-BBODE-LSSVM	8.77114 *	9.34361 *	11.15465 *	9.87993 *	10.61179 *	10.64809 *
EEMD-LSSVM	CEEMD-BBODE-LSSVM	5.63757 *	9.63785 *	8.88177 *	9.19687 *	9.92837 *	4.73004 *
CEEMD-LSSVM	CEEMD-BBODE-LSSVM	3.93133 *	3.29112 *	3.60436 *	2.21033 **	5.59378 *	1.98392 **
***D-M*** **Test**		**Oct.**					
**Benchmark Model**	**Target Model**	**PM_2.5_**	**PM_10_**	**O_3_**	**CO**	**NO_2_**	**SO_2_**
LSSVM	CEEMD-BBODE-LSSVM	5.26581 *	6.48092 *	9.63251 *	5.52022 *	9.57181 *	10.60434 *
EEMD-LSSVM	CEEMD-BBODE-LSSVM	7.64110 *	7.62847 *	10.65943 *	5.52252 *	7.21038 *	5.42034 *
CEEMD-LSSVM	CEEMD-BBODE-LSSVM	3.26291 *	2.27028 **	4.95977 *	1.48417	5.29233 *	1.99318 **

* Denotes the 1% significance level; ** Denotes the 5% significance level; *** Denotes the 10% significance level.

## Abbreviations

CTM	chemical transport model
MLR	multiple linear regression
ARIMA	integrated moving average model
GRNN	general regression neural network
LSSVM	least squares support vector machine
SVM	support vector machine
AHP	analytical hierarchical process
EMD	empirical mode decomposition
EEMD	ensemble empirical mode decomposition
CEEMD	complementary ensemble empirical mode decomposition
IMF	intrinsic mode function
DE	differential evolution
BBO	biogeography-based optimization
PDF	probabilistic distribution function
CDF	cumulative distribution function
AW	average width
CP	coverage probability
AQI	air quality index
MAE	mean absolute error
MAPE	mean absolute percentage error
RMSE	root mean square error
*R*^2^	goodness of fit
Std.	standard deviation

## Appendix A

### Appendix 1. The PDF and CDF Functions for Weibull, Gamma, Lognormal, Log-Logistic, Inverse Gaussian

**Table A1:** The PDF and CDF of five kinds of distributions.

Distribution	PDF/CDF	Parameters
Weibull	$f(x;a,b)=\frac{b}{a}{\left(\frac{x}{a}\right)}^{b-1}\exp[-{\left(\frac{x}{a}\right)}^{b}],x\geq 0$	*a* > 0 scale parameter *b* > 0 shape parameter
	$F(x;a,b)=1-\exp[-{\left(\frac{x}{a}\right)}^{b}]$	
Gamma	$f(x;a,b)=x^{a-1}\exp(-\frac{x}{b})/b^{a}\Gamma (a),x>0$	*a* > 0 shape parameter *b* > 0 scale parameter
	$F(x;a,b)=\frac{1}{b^{a}\Gamma (a)}\int_{0}^{x}t^{a-1}\exp(-\frac{x}{b})dt,x>0$	
Lognormal	$f(x;a,b)=\frac{1}{xa\sqrt{2\pi }}exp(-\frac{{\left(Inx-b\right)}^{2}}{2a^{2}}),x>0$	*a* > 0 scale parameter *b* > 0 location parameter
	$F(x;a,b)=\frac{1}{a\sqrt{2\pi }}\int_{0}^{x}\frac{1}{t}\exp(-\frac{{\left(Int-b\right)}^{2}}{2a^{2}})dt$	
Log-logistic	$f(x;a,b)=\frac{(b/a){\left(x/a\right)}^{b-1}}{{\left(1+{\left(x/a\right)}^{b}\right)}^{2}},x>0$	*a* > 0 scale parameter *b* > 0 shape parameter
	$F(x;a,b)=\frac{x^{b}}{a^{b}+x^{b}}$	
Inverse Gaussian	$f(x;a,b)={\left[\frac{b}{2\pi x^{3}}\right]}^{1/2}\exp\left[\frac{-b{\left(x-a\right)}^{2}}{2a^{2}x}\right],x>0$	*a* > 0 scale parameter *b* > 0 shape parameter
	$F(x;a,b)=\Phi \left[\sqrt{\frac{b}{x}}(\frac{x}{a}-1)\right]+\left[\exp(\frac{2b}{a})\right]\Phi \left[-\sqrt{\frac{b}{x}}(\frac{x}{a}+1)\right]$	

### Appendix 2. The Test Functions in This Paper for BBO and BBODE Algorithm

**Table A2:** Test functions.

Function Name	Test Function	Variable Domain	Global Optimum
Sphere	$f\left(x\right)=\sum_{i=1}^{d}x_{i}^{2}$	$x_{i}\in \left[-100,100\right]$	$f_{min}\left(0,0,0\cdots 0\right)=0$
Rosenbrock	$f\left(x\right)=\sum_{i=1}^{d-1}\left[100{\left(x_{i}^{2}-x_{i+1}\right)}^{2}+{\left(x_{i}-1\right)}^{2}\right]$	$x_{i}\in \left[-30,30\right]$	$f_{min}\left(1,1,1\cdots 1\right)=0$
Rastrigin	$f\left(x\right)=\sum_{i=1}^{d}\left(x_{i}^{2}-10cos\left(2\pi x_{i}\right)+10\right)$	$x_{i}\in \left[-5.12,5.12\right]$	$f_{min}\left(0,0,0\cdots 0\right)=0$
Shaffer	$f(x)=0.5+\frac{{\left(sin\sqrt{\sum_{i=1}^{d}{x_{i}}^{2}}\right)}^{2}-0.5}{{\left(1+0.001\sum_{i=1}^{d}{x_{i}}^{2}\right)}^{2}}$	$x_{i}\in \left[-100,100\right]$	$f_{min}\left(0,0,0\cdots 0\right)=0$
Griewank	$f(x)=\frac{1}{4000}\sum_{i=1}^{d}{x_{i}}^{2}-\prod_{i=1}^{d}cos(\frac{x_{i}}{\sqrt{i}})+1$	$x_{i}\in \left[-600,600\right]$	$f_{min}\left(0,0,0\cdots 0\right)=0$
Ackley	$\begin{array}{l} f(x)=-a\exp\left(-b\sqrt{\frac{1}{n}\sum_{i=1}^{d}{x_{i}}^{2}}\right)-\exp\left(\frac{1}{n}\sum_{i=1}^{d}cos(2\pi x_{j})\right)+a+e\\ a=20,b=0.2,e=2.7128 \end{array}$	$x_{i}\in \left[-32,32\right]$	$f_{min}\left(0,0,0\cdots 0\right)=0$

### Appendix 3. Pseudo-Code of the BBODE Algorithm

The detailed pseudo-code of the BBODE algorithm used in this paper can be summarized as follows:

***Parameters***
*t*: the number of iteration *Iter_Max*: the maximum number of iteration
*n*: the maximum number of species *size*: the size of population
*rand*: the random number in [0,1] *V_i_*: the mixed hybrid operator
*MaxX_i_*: the maximum individual *MinX_i_*: the minimum individual
*C*: the probability of mutation *F*: difference operator
1 /* Parameter setup */
2 /* Initialize population *P_i_* */
3 /* Compute the fitness function *F_i_* of each habitat, sort *F_i_* */
4 $F_{i}=\mathrm{MSE}(\left|y_{real}-{\hat{y}}_{forecast}\right|)$
5 /* Obtain elitist population */
6 /* Initialize probability of population in habitat */
7 **FOR** *t* < Iter_Max **DO**
8 **IF** *t* is odd **THEN**
9 /* Compute the number of population *k* */
10 /* Compute the rate of immigration *λ_i_* and emigration *μ_i_* for each habitat */
11 $\lambda_{i}=0.5I(1+cos(\frac{k\pi }{n}))$; $\mu_{i}=0.5E(1-cos(\frac{k\pi }{n}))$
12 /* Normalize the immigration rate *λ_scale_* */
13 $\lambda_{scale}=\lambda_{lower}+(\lambda_{upper}-\lambda_{lower})*(\lambda_{i}-\min(\lambda_{i}))/(\max(\lambda_{i})-\min(\lambda_{i}))$
14 /* Operation of migration */
15 Transform new information to habitat *i*
16 **ELSE**
17 **FOR** *i* = 1:*size*
18 Choose indexes *r_1_* ≠ *r_2_* ≠ *i*
19 /* Generate difference operator */
20 $V_{i}=P_{i}+F*(P_{i}(1)-P_{i})+F*(P_{i}(r_{1})-P_{i}(r_{2}))$
21 **IF** *rand* ≤ *C* **THEN**
22 /* Mutation operation */
23 $V_{i}=MinX_{i}+(MaxX_{i}-MinX_{i})*rand$
24 **END IF**
25 **END FOR**
26 **END IF**
27 **END FOR**
28 /* Deassign for samples beyond the range */
29 /* Deassign for the same sample */
30 /* Compute fitness *F_i_* for new population and sort *F_i_* */
31 Obtain optimal solution
32 Postprocess results and visualization
